# Planned percutaneous nephrolithotomy in patients who initially presented with urosepsis: Analysis of outcomes and complications

**DOI:** 10.1080/2090598X.2021.2002635

**Published:** 2021-11-26

**Authors:** Ahmed Fahmy, Karim Saad, Wael Sameh, Omar Elgebaly

**Affiliations:** Alexandria Faculty of Medicine, Alexandria University, Alexandria, Egypt

**Keywords:** Percutaneous nephrolithotomy, urosepsis, outcomes, complications

## Abstract

**Objective:**

To compare the outcomes and complications of planned percutaneous nephrolithotomy (PCNL) in patients with a prior urosepsis episode to those without.

**Patients and Methods:**

We recorded patients who presented initially with obstructive urosepsis, as identified by systemic inflammatory response syndrome and obstructing kidney stones. We compared the surgical outcomes and complications among those patients who had planned PCNL after control of prior urosepsis with urgent decompression and antibiotics (Group A) to a group who presented for PCNL with no previous history of a septic presentations (Group B). A 1:1 matched-pair analysis was performed using four parameters (age, gender, body mass index, and American Society of Anesthesiologists classification) to eliminate potential allocation bias. Primary outcomes included were stone-free rate (SFR) and complication rate. Secondary outcomes included were operative time, estimated blood loss, and duration of postoperative hospital stay.

**Results:**

A total of 80 patients underwent PCNL (48 male and 32 females) divided equally between both treatment groups, with a mean (interquartile range) age of 47 (19–75) years. There were no differences in demographic data or stone characteristics between both groups. Both groups had comparable SFRs (92.5% vs 97.5%, *P* = 0.212) and mean operative time (77 vs 74 min, *P* = 0.728) (Table 2). Patients in Group A had a significantly higher overall complications rate (35% vs 10%, *P* = 0.03) . There were no postoperative mortalities and the mean length of hospital stay was significantly longer in Group A patients compared to group B (4.2 vs 1.5 days, *P* = 0.042).

**Conclusions:**

: Planned PCNL after decompression for urolithiasis-related sepsis has comparable operative time and SFR but higher complication rates and longer postoperative hospital stay. This is critical in counselling patients prior to definitive treatment of kidney stones after urgent decompression for urosepsis and for adequate preoperative planning and preparation.

**Abbreviations:** ASA: American Society of Anesthesiologists; BMI: body mass index; ICU: intensive care unit; IQR: interquartile range; KUB: plain abdominal radiograph of the kidneys, ureters and bladder; PCN: percutaneous nephrostomy; PCNL: percutaneous nephrolithotomy; SFR: stone-free rate; URS; ureteroscopy; US: ultrasonography

## Introduction

Percutaneous nephrolithotomy (PCNL) is the minimally invasive procedure of choice for the management of large-volume and complex kidney stones because of its high efficacy and safety yielding high stone-free rates (SFRs) [1]. Early diagnosis and prompt urgent management of urosepsis related to obstructive urolithiasis is pivotal for improved clinical outcomes [2].

Renal decompression through percutaneous nephrostomy (PCN) or retrograde JJ stent is almost always the first step in managing patients presenting with an obstructing stone and urosepsis followed by definitive treatment for the stone at a later stage, once the patient’s general condition improves and sepsis controlled [3,4]. However, clinicians should counsel patients that treatment of the stones may initially be more complex, with increased hospital stay and stent time [5].

The influence of prior urosepsis on perioperative outcomes of PCNL has not been thoroughly investigated, and whether prior urosepsis is associated with higher intra- and postoperative complications remains unclear. The aim of the present study was to compare the outcomes and complications of planned PCNL in patients with a prior urosepsis episode to those without.

## Patients and methods

A retrospective review of the data of patients who underwent PCNL procedures for kidney stones in our Urology Department between August 2016 and March 2020 was carried out. Inclusion criteria were patients aged >18 years who had kidney stones. Patients with any contraindication for PCNL (such as uncorrected coagulopathy or UTI), solitary kidney or renal impairment were excluded from the study. The study was approved by the local Ethics Committee. Informed consent was obtained from all individual participants included in the study.

We recorded patients who presented initially with obstructive urosepsis, as identified by systemic inflammatory response syndrome and obstructing kidney stones [6]. We compared the surgical outcomes and complications among those patients who had planned PCNL after control of prior urosepsis with urgent decompression and antibiotics (Group A) to a cohort group who presented for PCNL with no previous history of a septic presentations (Group B).

Demographic data including patient age, sex, body mass index (BMI), American Society of Anesthesiologists (ASA) classification, stone characteristics, intraoperative events, outcomes of the procedures, and postoperative hospital stay were collected by reviewing hospital medical records.

The stone length was calculated according to the longest diameter, and the stone burden was calculated by multiplying its length by its width. The operative duration was calculated from the time of initial cystoscopic ureteric catheter placement until securing the nephrostomy tube. Stone-free status was defined as no residual fragments of ≥4 mm on plain abdominal radiograph of the kidneys, ureters and bladder (KUB) and ultrasonography (US) of the urinary tract after 1 month. The haemoglobin deficit was the difference between the preoperative level and its level 12-h postoperatively. All procedures were performed or supervised by a single senior endourologist with a high expertise in percutaneous renal surgery to ameliorate potential performance bias when procedures are not performed in a uniform way.

A 1:1 matching of the patients based on their propensity scores was performed, to eliminate potential allocation bias, using a logistic regression model, with prior urosepsis as the dependent variable in relation to the following baseline characteristics: age, sex, BMI, ASA classification. We did not include stone characteristics in the matching criteria, because this would have limited the number of patients included in the study.

### Preoperative evaluation

Upon complete urosepsis control in Group A patients, a planned elective PCNL was scheduled for these patients. On admission for elective PCNL, patients were re-evaluated regarding their fitness for the endoscopic intervention at a dedicated pre-assessment clinic in conjunction with the anaesthesia team. Preoperative and admission-related data included urine analysis, urine culture, blood culture, full blood count, biochemistry study, renal US, KUB, and whole abdominal CT were obtained and evaluated upon admission.

All microbiology results were re-assessed and checked for patients in both groups before the endoscopic intervention. Patients with symptomatic and/or culture-confirmed UTI and high-risk patients with low ASA classification received an appropriate course of antibiotics, at least 1 week prior to the procedure. In case of negative urine culture, a single perioperative prophylactic dose of antibiotics was administrated before the endoscopic intervention. This was performed under the supervision of the microbiology team.

### Intervention

All procedures were performed under general anaesthesia. After a cystoscopic retrograde ureteric catheter was fixed, with the patient in the lithotomy position, the patient was placed in the prone position. The skin was punctured at the posterior axillary line under multidirectional C-arm fluoroscopic guidance (BV Pulsera, Philips Medical Systems, Eindhoven, the Netherlands) and posterior calyx was entered with 18-G translumbar angioplasty needle (Boston Scientific, Natick, MA, USA). In some cases (16 patients in Group A), access to the pelvicalyceal system was obtained via the nephrostomy tube tract.

Before beginning tract dilatation, all planned tracts were established, working and safety guidewires were secured inside the pelvicalyceal system. Dilatation of the tract was achieved with coaxial telescopic dilators (Karl Storz Endoskope, Tuttlingen, Germany). A 30-F Amplatz sheath (Boston Scientific Corp., Natick, MA, USA) and a 27-F semi-rigid nephroscope (Karl Storz Endoskope) were used for all procedures. Pneumatic lithotripsy (Swiss Lithoclast, EMS, Nyon, Switzerland) was utilised for stone disintegration. Stone-free status was confirmed intraoperatively both endoscopically and fluoroscopically. A nephrostomy tube (22 F) was placed at the end of the procedure.

### Postoperative evaluation and follow up

Renal US and KUB were performed before removal of the nephrostomy tube for detection of residual stones. Any residual stone of >10 mm accessible through the existing nephrostomy tracts was treated by a second session of PCNL, while ESWL was used for residual fragments of 4–10 mm. All patients underwent renal US after 1 month for confirmation of stone-free status, which was defined as no residual fragments of ≥4 mm.

### Outcome measures

Primary outcomes included were SFR and complication rate. The SFR was defined as the absence of any residual stones of ≥4 mm on postoperative imaging performed 1 month after PCNL. Postoperative complications were assessed and graded according to the modified Clavien–Dindo classification [7]. Major complications were defined as Clavien–Dindo Grade III–V. All complications were defined as those occurring within the first month postoperatively or before discharge, whichever the longer time frame. Secondary outcomes included operative time, estimated blood loss, and duration of postoperative hospital stay.

### Statistical methods

Continuous variables were expressed as mean (± SD). If the parametric test assumptions were met, the Student’s *t*-test was used for comparing between the two groups, while the Mann–Whitney *U*-test was used if the parametric test assumptions were not met. Categorical variables were expressed as frequency (*n*) and percentage (%) and compared with chi-squared or Fisher’s exact test. Independent predictors for SFR or complications were evaluated using uni- and multivariate logistic regression to control for baseline differences between the two groups. A *P* < 0.05 was considered statistically significant and all the analyses were performed using R software version 3.6.0. (R Core Team [2019]. R: A language and environment for statistical computing. R Foundation for Statistical Computing, Vienna, Austria. URL https://www.R-project.org/).

## Results

A total of 80 patients underwent PCNL (48 male and 32 females) divided equally between both treatment groups, with a mean (interquartile range [IQR]) age of 47 (19–75) years. Table 1 presents the demographic characteristics of the studied patients in both groups.Table 2

**Table 1.** Patients’ characteristics

Characteristic	Group A (*N* = 40)	Group B (*N* = 40)	*P*
Age, years, median (IQR)	48 (19–76)	52 (19–80)	0.152
Gender, male/female, *n*	22/18	26/14	0.751
BMI, kg/m^2^, mean (SD)	28.49 (2.69)	27.13 (5.24)	0.503
Stone sizes, mm, mean (SD)	26.42 (4.54)	27.19 (6.25)	0.728
Stone side, left/right, *n*	13/27	15/25	0.320
Stone burden, mm^2^, mean (SD)	376.68 (17)	412.46 (81)	0.654
Stone number, single/multiple	16/24	20/20	0.140

**Table 2.** Clinical outcomes

Outcome	Group A (*N* = 40)	Group B (*N* = 40)	*P*
SFR, *n/N* (%)	37/40 (92.5)	39/40 (97.5)	0.212
Operative time, min, mean (range)	77 (44–116)	74 (48–110)	0.728
Overall complications, *n/N* (%)	14/40 (35)	4/40 (10)	0.03*
High-grade complications, *n* (%)	5 (12.5)	0	0.001*
Length of hospital stay, days, mean (range)	4.2 (1–9)	1.5 (1–3)	0.042*
Postoperative admission toICU, *n*	2	0	0.152

*Statistically significant at *P* < 0.05.

Among patients in Group A, emergency decompression was achieved via PCN in 28 (70%) and JJ stent placement in 12 (30%). In all, 13 patients (32.5%) required admission to the intensive care unit (ICU) at initial presentation of urosepsis. The median (IQR) time interval between initial PCN or JJ stent during the urosepsis episode and their elective PCNL was 37 (10–92) days. A positive urine culture on presentation was obtained in 36 patients (90%) who initially presented with urosepsis. The organisms identified were *Escherichia coli* (17 patients, 67.5%), *Pseudomonas* (five, 12.5%), *Klebsiella* (three 3,7.5%) and *Proteus* (one, 2.5%).

There were no differences in demographic data or stone characteristics between the groups. Both groups had comparable SFRs (92.5% vs 97.5%, *P* = 0.212) and mean operative time (77 vs 74 min, *P* = 0.728), with no statistically significant difference. There was no need for second-look nephroscopy in any patient in either of the treatment groups; however, three patients (two in Group A and one in Group B) required ESWL for complete stone clearance. Patients in Group A had significantly higher overall complications rate (35% vs 10%, *P* = 0.03). Intraoperative complications included three patients in Group A with significant bleeding necessitating intraoperative blood transfusion, while no patient in Group B needed blood transfusion.

Subgroup analysis regarding methods of drainage revealed no significant difference between perioperative outcomes of those who had nephrostomy tube vs those who had a JJ stent for relief of urosepsis in Group A, including SFR (96.4% vs 83.3%, *P* = 0.76), operative times (72.7 vs 79.2 min, *P* = 0.26), length of hospital stay (2.8 vs 3.8 days, *P* = 0.1), and complications rate.

Table 3 shows postoperative complications included fever (four patients in Group A and two in Group B), two patients developed sepsis postoperatively needing ICU admission in Group A. Mild gross haematuria in two patients (5%) in each group, which was managed conservatively by bed rest, intravenous fluids, and nephrostomy tube clamping. One patient presented by severe gross haematuria needed selective angioembolisation for symptomatic arteriovenous fistula confirmed by CT angiography in Group A. Persistent urinary leakage from the nephrostomy site was noted in two patients in Group A, which was managed with endoscopic retrograde JJ stent placement. There were no postoperative mortalities in our study and the mean length of hospital stay was significantly longer in Group A patients compared to group B (4.2 vs 1.5 days, *P* = 0.042).

**Table 3.** Perioperative complications (Clavien–Dindo grading)

Clavien–Dindo Grade	Description	Group A, *n* (%)	Group B, *n* (%)
I	Fever	4 (10)	2 (5)
II	Blood transfusion Haematuria	3 (7.5) 2 (5)	0 2 (5)
IIIA IIIB	Angioembolisation Persistent urinary leakage	1 (2.5) 2 (5)	0 0
IV	Sepsis	2 (5)	0
V	Death	0	0

Multivariate logistic regression analysis showed that stone size, side, burden, and number were not independent predictors of stone-free status or developing complications after PCNL procedure. Initial presentation with urosepsis was statistically significantly related to the complication rate in multivariate models (odds ratio 3.251, 95% CI 1.829–8.654; *P* = 0.001).

## Discussion

Urosepsis due to an obstructing stone must be managed by urgent decompression of the obstructed kidney, aggressive fluid resuscitation with haemodynamic support associated with controlling the source of the infection by an appropriate course of antibiotics [8,9]. This should be followed by definitive management of the stone, once the infection has resolved and the patient’s general conditions have improved [3,4,8].

It was assumed that, patients undergoing planned endourological approaches after prior urosepsis decompression were more likely to have more complex procedure and a higher complication rate [10,11]. Prolonged antibiotic administration, longer postoperative hospital stay, and auxiliary procedures may also be needed. This suggests that patients with prior urosepsis, even those adequately managed in the emergency phase, are more prone to complications after the elective endourological treatment [12–14]. A possible explanation for this may be attributed to the residual effect of inflammatory changes induced by urosepsis on renal parenchyma and microvasculature [15]. In addition, patients’ frailty, associated comorbidities in patients with prior urosepsis and the more prevalence of septic foci including infection stones in the urinary tracts are other potential causes among this group of patients [16,17].

Few publications have investigated the impact of prior urosepsis as an independent risk factor on surgical outcomes and complications after definitive endourological approaches. A retrospective matched-pair comparison between elective ureteroscopy (URS) and patients with prior urosepsis showed SFRs were similar between the two groups, but patients with history of prior sepsis were more likely to have increased hospital stay, require prolonged antibiotic use, and have prolonged stent duration [18]. Another study reported the outcomes of elective URS stone treatment in 76 patients with prior sepsis and emergency drainage, the SFR was 97% and there was only one high-grade complication [5].

The optimal duration of antibiotic treatment and waiting time before definitive treatment after urgent decompression for patients who initially present with urosepsis have not been thoroughly investigated and determined [19]. In the present study, patients who presented with sepsis were treated initially with decompression either by PCN or endoscopic JJ insertion, intravenous antibiotics were administrated for a median period of 7 days (up to 14 days) followed by oral antibiotics for 1–2 weeks.

Patients were treated definitively by PCNL after a median (IQR) period of 37 (10–92) days from initial decompression of sepsis. This was influenced by multiple factors including the severity of initial presentation, presence of comorbidities, clinical response to treatment after decompression and antibiotics, subsequent anaesthetic assessment of fitness for their elective procedure, waiting list time, and patient choice. The appropriate waiting time for definitive management after initial decompression and its potential impact on success and complications rate could not be elucidated from our present study due to the relatively small number of patients and few complications. In addition, many of these cases were initially managed outside our hospital by renal decompression for urosepsis control and presented to us later for definitive PCNL procedure.

In the present study, the SFR was similar between the two treatment groups. History of prior urosepsis did not affect the SFR as long as the technical steps are strictly followed, PCNL can still be performed with a comparable success rate.

Patients in Group A had a higher overall complication rate (35% vs 10%) and severity of complications compared to Group B. None of patients in Group B developed a high-grade complication (Grade III–V); however, five patients in Group A had high-grade complications. This substantial difference can be explained to some extent by urosepsis induced effect on renal parenchyma including tissue inflammation, ischaemia, vascular damage, and the presence of uncleared septic foci inside the urinary system resulting in an increase in the risk of intra- and postoperative complications. The mean postoperative hospital stay was 4.2 and 1.5 days in groups A and B, respectively (*P* = 0.042). Postoperative adverse events were more prevalent among patients in Group A and the time needed for their appropriate management may account for the more prolonged postoperative hospital stay.

In the present study, matching of patients was successfully performed based on age, gender, BMI, and ASA classification for better patient characteristics adjustment. Our results revealed that prior urosepsis is predictive of adverse perioperative outcomes in patients requiring elective PCNL after urosepsis control and was associated with higher grades of complications.

Considering the results of the present study, the following limitations should be taken into account. The first limitation is its retrospective nature, representing a single centre experience, which implicates potential confounding. To control for this, we matched patients based on propensity scores. Second, the relatively small number of patients included, which precluded the meaningful analysis of other independent predictors for clinical outcomes or complication rates, especially with the relatively small number of outcomes. Finally, we were not able to study the difference in clinical outcome and complications between both groups based on the type of stone composition, as stone analysis was not available in our data. It was demonstrated that infection stone composition could negatively affect the overall complication rate with previous sepsis [17].

However, the advantage of the present study is its ability to demonstrate higher complication rates and longer postoperative hospital stay associated with elective PCNL after treatment of urosepsis. This may help in counselling a selected group of patients with a higher risk of intra- and postoperative complications, thus could potentially improve surgical outcomes through adequate preoperative planning and preparation. Future prospective studies on larger numbers of patients are needed to confirm the findings of the present study.

## Conclusions

Planned PCNL after decompression for urolithiasis-related sepsis has comparable operative time and SFR but higher complication rates and longer postoperative hospital stays. This is critical in counselling patients prior to definitive treatment of kidney stones after urgent decompression for urosepsis and for adequate preoperative planning and preparation.
